# Reported outcomes in phase II studies of newly-diagnosed pulmonary tuberculosis

**DOI:** 10.1186/1745-6215-16-S1-P7

**Published:** 2015-05-29

**Authors:** Laura J Bonnett, Geraint Davies

**Affiliations:** 1 Department of Biostatistics, Institute of Translational Medicine, University of Liverpool, Liverpool, Merseyside, L69 3GA, UK; 2 Department of Clinical Infection, Microbiology & Immunology, Institute of Infection and Global Health, University of Liverpool, Liverpool, Merseyside, L69 7BE, UK

## Background

Tuberculosis (TB) remains a major killer amongst infectious diseases. Current treatment involves a four-drug regimen for at least six months. New drugs and regimens are required to shorten treatment duration, reduce toxicity and combat drug resistance however the optimal methodology to define the critical path for these regimens is not well-defined.

We undertook a literature review to summarise outcomes reported in phase II trials of patients with newly-diagnosed pulmonary TB to determine the necessity for a core outcome set in TB.

## Methods

Relevant studies were identified by searching MEDLINE, EMBASE, LILACs and reference lists of included studies. All reported outcomes were considered.

## Results

49 included phase II studies presented data on 24 historic drug combinations. The most commonly reported outcome (35 studies) was early bactericidal activity (EBA) although there was disagreement regarding a definition. The majority of studies defined EBA as the fall (sometimes mean rate of change) in log_10_ colony forming units (CFU) per ml sputum over various time points. Related outcomes such as CFU count were sometimes reported in preference (12 studies). Other popular outcomes included number of positive (20 studies) or negative cultures (4 studies) at selected time points while in others time to culture negativity was favoured (3 studies). In all cases there was disagreement as to which time points should be chosen with options ranging from two days to eight weeks. Results for novel drug combinations are currently being analysed and will be presented.

## Conclusions

There is large variation in the outcomes reported across phase II studies in TB. To utilise results of multiple studies, and thus identify the best treatment regimens for TB, a core outcome set needs to be developed. This would enable trial results to be more easily compared and combined, potentially leading to improved treatment strategies for patients with TB.

